# Bloodstream infection due to *Escherichia coli* in liver cirrhosis patients: clinical features and outcomes

**DOI:** 10.18632/oncotarget.23200

**Published:** 2017-12-13

**Authors:** Bo Tu, Jingfeng Bi, Dan Wu, Peng Zhao, Lei Shi, Yangxin Xie, Xin Zhang, Zhe Xu, Suxia Liu, Xinhua Wang, Xiaoxi Li, Fusheng Wang, Enqiang Qin

**Affiliations:** ^1^ Center for Infectious Disease, Beijing 302 Hospital, Beijing 100039, China; ^2^ Center for Clinical Research Management, Beijing 302 Hospital, Beijing 100039, China; ^3^ Center for Liver Failure, Beijing 302 Hospital, Beijing 100039, China; ^4^ Center for Obstetrics and Gynecology, Beijing 302 Hospital, Beijing 100039, China; ^5^ Center for Clinical Laboratory, Beijing 302 Hospital, Beijing 100039, China

**Keywords:** liver cirrhosis, bloodstream infection, MELD, hospital-acquired BSI, ESBL

## Abstract

**Objectives:**

The study aimed to investigate the clinical characteristics and antibiotic management, as well as independent indicators for survival within 30 days for Escherichia coli bloodstream infection (BSI) in liver cirrhosis.

**Results:**

Hospital-acquired BSI accounted for 60.07%, with prolonged hospital stay (*P* = 0.000). The prevalence of Extended Spectrum Beta-Lactamases (ESBL) producing bacteria was 48.26%, which correlated with ICU admission (*P* = 0.015) and high model for end-stage liver disease (MELD) score at onset of BSI (*P* = 0.035). Moreover, ESBL producing pathogens showed a high resistant to the common antibiotic families and 27.5% pathogens were confirmed as multidrug-resistant (MDR). MDR infection was significantly correlated with ESBL production, ICU admission, inappropriate empiric therapy, resistance to firstly selected antibiotic, and infection duration (*P* < 0.05 for all). In addition, appropriate empiric therapy within 48 h (HR = 2.581, 95% CI = 1.166–5.715), ICU admission (HR = 4.434, 95% CI = 2.130–8.823), HE (HR = 2.379, 95% CI = 1.115–5.073) and final MELD (HR = 1.074, 95% CI = 1.044–1.106) were independent indicators for 30-day mortality.

**Materials and Methods:**

The clinical data were collected from 288 eligible patients, and compared according to survival status and sites of infection acquisition. Drug resistance was recorded according to ESBL. In addition, cox regression analysis model was applied to evaluate the risk factors for 30-day mortality.

**Conclusions:**

ESBL production can promote resistance to antibiotics in Escherichia coli. Antibiotic regimens, ICU admission, HE and MELD score can help identify the risk individuals who will benefit from the improved therapeutic regimens.

## INTRODUCTION

Liver cirrhosis is a troublesome problem for public health worldwide, with high occurrence rate and mortality [1]. Cirrhosis is an advance stage of chronic liver disease, due to chronic infection with hepatitis virus, alcohol abuse, fat accumulation, autoimmune and metabolic alterations [2, 3]. Various complications are frequently observed in cirrhosis patients, such as acute kidney injury, portal vein thrombosis, variable abdominal pain [4, 5]. Among the complications, bacterial infection is a leading cause for death [6]. Unfortunately, patients with liver cirrhosis are more easily to be infected by pathogens due to impaired immunity, consequently poor outcomes [7–9].

Bloodstream infection (BSI) is a serious problem in many hospitalized patients, and is referred as being primary without obvious infection source, or secondary, arising as a complication of infection elsewhere (such as pneumonia, urinary tract, skin and soft tissues, intra-abdominal, device-related, etc) [10]. BSI is a common complication in liver cirrhosis [11]. It was reported that the frequencies of BSI in cirrhosis patients were 10 times higher than that in the non-cirrhosis group [12]. Two major reasons may lead to the high occurrence of BSI in cirrhosis patients: dysregulated intestinal bacterial translocation and cirrhosis associated immune dysfunction (CAID) [13]. The abnormal intestinal barrier permeability, overgrowth of small intestinal bacterial, and immune dysfunction may promote the bacteria into the bloodstream, leading to BSI [14, 15]. BSI is significantly associated with prolonged hospital stay, faster development of liver disease, and low survival rate [7, 16]. A population-based cohort study indicated that BSI was a predictor for mortality in liver cirrhosis patients [17] The most common pathogens for BSI include gram-negative entericbacilli, anaerobes, and *Enterococcus spp.* [13]. In the previous studies, several studies reported the clinical characteristics of BSI patients. However, few studies described the specific characteristics of *Escherichia coli* BSI patients.

Timely and appropriately empirical antibiotic treatments are important for survival among BSI patients. Growing evidences have indicated that inappropriate antibiotic regimens are associated with increased mortality [18, 19]. Until now, the empirical antibiotics are still effective for infection treatments. However, its failure rate is increasing due to the growing prevalence of multidrug-resistant multidrug resistant (MDR) bacteria. MDR is defined as acquired non-susceptibility to at least one agent in three or more antimicrobial categories [20]. There are several common MDR, including extended-spectrum β-lactamase-producing bacteria (ESBL), methicillin-resistant *Staphylococcus aureus* (MRSA), etc [21]. ESBL productions is one of the major reasons for antibiotic resistance in *Escherichia coli*. Therefore, to investigate the association between ESBL status and clinical characteristics in liver cirrhosis patients presenting *Escherichia coli* BSI will be helpful to guide empirical therapy.

In the present study, we aimed to investigate the clinical characteristics and antibiotic management, as well as independent risk factors for *Escherichia coli* BSI in liver cirrhosis patients. The liver disease of the patients were evaluated by Child-Pugh and model for end-stage liver disease (MELD) scores. We investigated the clinical characteristics of patients according to ESBL status of pathogens and survival status within 30 days after BSI diagnosis as well as sites of acquisition infection.

## RESULTS

### Baseline characteristics of the study population

There were 288 eligible patients in the present study, with average age of 51.95 ± 11.71 years. The study population included 204 males and 84 females and their mean times for hospital admission was 2.76 ± 3.29. Among the recruited patients, 213 (73.96%), 29 (10.07%), 32 (11.11%) and 14 (4.86%) patients were diagnosed with viral cirrhosis, alcoholic cirrhosis, autoimmune cirrhosis cases, and cryptogenic cirrhosis, respectively. The majority of the patients were classed as Child-Pugh class B (98, 34.03%) and C (171, 59.37%), while only few of them were diagnosed with class A (19, 6.60%). 45 of the patients were admitted to intensive care unit (ICU) ward (15.63%) (Table 1).

**Table 1:** Baseline characteristics of the collected patients

Characteristics	Patients (*n* = 288, %)
Age (years)	51.95 ± 11.71
Gender (male/female)	204 (70.83)/84 (29.17)
Average times for hospital admission (times)	2.76 ± 3.29
Clinical characteristics of the patients within 2 years before admission	
Frequencies of SBP (mean ± SD)	0.25 ± 0.70
Frequencies of pneumonia (mean ± SD)	0.06 ± 0.31
Frequencies of septicemia (mean ± SD)	0.04 ± 0.21
Admission condition	
ICU admission (yes/no)	45 (15.63)/243 (84.37)
Pathogenesis of cirrhosis	
Viral	213 (73.96)
Alcoholic	29 (10.07)
Autoimmune	32 (11.11)
Cryptogenic	14 (4.86)
Combined with hepatocellular carcinoma (yes/no)	69 (23.96)/219 (76.04)
Child-Pugh	
A	19 (6.60)
B	98 (34.03)
C	171 (59.37)
Mean MELD value	16.84 ± 7.99
Complication during hospital stay	
HE (yes/no)	57 (19.79)/231 (80.21)
SBP (yes/no)	141 (48.96)/147 (51.04)
Pneumonia (yes/no)	26 (9.03)/262 (90.97)
Septic shock (yes/no)	53 (18.40)/235 (81.60)
BSI data	
Unknown cause	228 (79.17)
Digestive tract	31 (10.76)
Vascular intervention operation	13 (4.51)
Skin intervention operation	13 (4.51)
HCC	3 (1.04)
ESBLs (+/−)	139 (48.26)/149 (51.74)
Hospital-acquired BSI (yes/no)	173 (60.07)/115 (39.93)
MELD at BSI	15.79 ± 7.63
Δ MELD (at BSI-baseline) (mean ± SD)	−1.04 ± 4.34
Susceptibility to the firstly selected antibiotic (sensitive/resistant)	219 (76.04)/69 (23.96)
Shiver (yes/no)	85 (29.51)/203 (70.49)
Chill (yes/no)	51 (17.71)/237 (82.29)
Maximum body temperature (°C)	39.16 ± 0.68
Clinical outcomes	
Average duration in hospital (days)	23.93 ± 16.99
Duration of infection (days)	15.20 ± 11.69
Non-survivors	34 (11.81)
Improved	186 (64.58)
Invalid	68 (23.61)
MELD	16.12 ± 9.96
Appropriate empiric therapy within 48 h	
Yes	253 (87.85)
No	35 (12.15)

Note: ICU: Intensive care unit; MELD: Model for end-stage liver diseases; HE: Hepatic encephalopathy; SBP: Spontaneous bacterial peritonitis; BSI: Bloodstream infections; ESBLs: Extended spectrum beta-lactamases; HCC: Hepatocellular carcinoma.

In addition, 60.07% (173/288) of the study subjects were diagnosed with hospital-acquired BSI. The source of BSI included primary infection with unknown cause (228, 79.17%), and secondary infection with digestive tract (31, 10.76%), vascular intervention operation (13, 4.51%), skin intervention operation (13, 4.51%) and HCC (3, 1.04%). 139 (48.26%) of the collected patients were diagnosed with ESBL positive (ESBL^+^), while 149 (51.74%) patients were confirmed with ESBL negative (ESBL^−^). The average infection time for the study subjects was 15.20 ± 11.69 days. 253 (87.85%) patients received appropriate empiric therapy within 48h after BSI diagnosis, while 35 (12.15%) patients were treated with inappropriate antibiotics. The average duration in hospital of the study subjects was 23.93 ± 16.99 days. 30 days after BSI diagnosis, the condition of 186 (64.85%) patients were significantly improved, while therapeutic approaches were invalid in 68 (23.61%) patients. Overall 34 patients were dead and the mortality was 11.81%. The clinical characteristics of the study population were listed in Table 1.

### Clinical effects of hospital-acquired BSI in cirrhosis patients with BSI

Among the collected patients, the prevalence of hospital acquired BSI was 60.07%. We evaluated the effects of sites of acquisition infection on clinical characteristics of the study population. Analysis results indicated that cirrhosis patients with hospital-acquired BSI had a longer hospital stay than those without hospital acquired BSI (*P* = 0.000). Moreover, the MELD score at the discharged was also significantly associated with hospital acquired BSI (*P* = 0.000) (Table 2).

**Table 2:** Comparison of clinical characteristics in the study population according to their acquisition sites of infections, 30-day survival status

Factors	Acquisition sites of infections			30-day survival status		
	Hospital-acquired BSI (*n* = 173)	Non hospital-acquired BSI (*n* = 115)	*P* value	Survivors (*n* = 254)	Non-survivors (*n* = 34)	*P* value
Child-Pugh			0.272			0.058
A	14	5		19	0	
B	54	44		90	8	
C	105	66		145	26	
ESBL			0.118			0.829
+	77	62		122	17	
-	96	53		132	17	
Hospital-acquired BSI			-			0.874
yes	-	-		153	20	
no	-	-		101	14	
ICU admission			0.315			0.000
yes	24	21		30	15	
no	149	94		224	19	
Pathogenesis of cirrhosis			0.728			0.554
viral	132	81		190	23	
alcoholic	16	13		25	4	
autoimmune	17	15		26	6	
cryptogenic	8	6		13	1	
HCC			0.662			0.715
yes	43	26		60	9	
no	130	89		194	25	
BSI source			0.496			0.006
unknown cause	137	91		206	22	
digestive tract	16	15		21	10	
vascular intervention operation	9	4		12	1	
skin intervention operation	8	5		12	1	
HCC	3	0		3	0	
HE			0.708			0.004
yes	33	24		44	13	
no	140	91		210	21	
SBP			0.202			0.621
yes	90	51		123	18	
no	183	64		131	16	
Pneumonia			0.873			0.000
yes	16	10		17	9	
no	157	105		237	25	
Septic shock			0.234			0.000
yes	28	25		38	15	
no	145	90		216	19	
Susceptibility to the firstly selected antibiotic			0.693			0.493
sensitive	133	86		61	10	
resistant	40	29		193	24	
Hospital stays (days)	29.22 ± 18.14	15.98 ± 11.12	0.000	24.29 ± 16.77	21.29 ± 18.60	0.335
Clinical outcomes			0.124			-
non-survivors	20	14		0	34	
improved	105	81		186	0	
invalid	48	20		68	0	
MELD at admission	17.48 ± 8.22	15.87 ± 7.57	0.094	16.23 ± 7.61	21.38 ± 9.31	0.000
MELD at onset of BSI	15.67 ± 7.3	15.98/ ± 8.14	0.735	15.28 ± 7.21	19.62 ± 9.52	0.002
Final MELD	17.83 ± 10.23	13.54 ± 8.98	0.000	15.02 ± 8.82	24.35 ± 13.69	0.000
Duration of infection (days)	14.96 ± 12.20	15.57 ± 10.93	0.663	12.29 ± 12.71	15.59 ± 11.52	0.122
Appropriate empiric therapy within 48h			0.138			0.003
yes	153	97		226	24	
no	17	18		28	10	

Note: ICU: Intensive care unit; MELD: Model for end-stage liver diseases; HE: Hepatic encephalopathy; SBP: Spontaneous bacterial peritonitis; BSI: Bloodstream infections; ESBLs: Extended spectrum beta-lactamases; HCC: Hepatocellular carcinoma; Final MELD: detected at discharged.

### Effects of ESBLs status on clinical characteristics based on study group

The patients were dived into two groups according to ESBL status of their isolates: ESBL^+^ (*n* = 139) and ESBL^−^ (*n* = 149). The prevalence of ESBL producing bacteria was 48.26%. We compared the clinical characteristics between the two groups. Analysis results indicated that ICU (*P* = 0.018) and MELD at onset of BSI (*P* = 0.035) were significantly associated with status of ESBL. In addition, we also found that liver cirrhosis patients infected by ESBL positive bacteria showed low sensitivity to firstly selected antibiotics (*P* = 0.017), moreover, ESBL infection was significantly correlated with inappropriate empiric therapy within 48 h (*P* = 0.049). Other factors were similar between the two groups (*P* > 0.05 for all) (Table 3).

**Table 3:** Effects of infection characteristics on clinical symptoms among the study population

Factors	ESBL status			MDR infection		
	ESBLs positive (*n* = 139)	ESBLs negative (*n* = 149)	*P* value	MDR positive (*n* = 55)	MDR negative (*n* = 145)	*P* value
Child-Pugh			0.724			0.424
A + B	55	62		21	64	
C	84	87		34	80	
ESBL status			-			0.000
positive	-	-		48	39	
negative	-	-		7	105	
Hospital-acquired BSI			0.118			0.774
yes	77	96		32	87	
no	62	53		23	57	
ICU admission			0.018			0.005
yes	29	16		16	18	
no	110	133		39	126	
Septic shock			0.898			0.052
yes	26	27		15	22	
no	113	122		40	122	
Susceptibility to the firstly selected antibiotic			0.017			0.025
sensitive	96	121		36	116	
resistant	43	28		19	28	
Clinical outcomes			0.973			0.840
Non-survivors	17	17		9	19	
improved	89	97		33	91	
invalid	33	35		13	34	
MELD at admission	16.27 ± 8.27	17.37 ± 7.72	0.242	16.36 ± 7.53	17.44 ± 8.35	0.406
MELD at onset of BSI	14.81 ± 7.53	16.71 ± 7.64	0.035	15.16 ± 7.98	16.17 ± 7.24	0.396
Final MELD	15.18 ± 9.95	16.99 ± 9.92	0.123	15.69 ± 9.70	17.23 ± 10.61	0.351
Duration of infection (days)	15.26 ± 11.13	15.15 ± 12.23	0.940	16.20 ± 12.21	12.07 ± 7.71	0.021
Appropriate antibiotics within 48h			0.049			0.011
yes	115	135		41	128	
no	24	41		14	16	

Note: MDR: Multi-drug resistance.

### Drug susceptibility analysis

In the present study, we compared drug susceptibility according to ESBL status of the isolated pathogens. The results demonstrated that the susceptibilities to cefoperazone (*P* = 0.000), ceftriaxone (*P* = 0.000), cefepime (*P* = 0.000), cefotaxime (*P* = 0.000), ceftazidime (*P* = 0.000), levofloxacin (*P* = 0.000), gatifloxacin (*P* = 0.022), piperacillin (*P* = 0.000), SMZCO (*P* = 0.000), aztreonam (*P* = 0.000), fosfomycin (*P* = 0.023), furadantin (*P* = 0.025), ticarcillin/clavulanate potassium (*P* = 0.016), and ampicillin (*P* = 0.000) were significantly influenced by ESBL status. On the other side, ESBL status were not correlated with resistance to cefoperazone-sulbactam, cefmetazon, meropenem, amikacin, minocycline, and piperacillin/tazobactam (*P* > 0.05 for all) (Table 4). It was worth noting that among the isolated bacteria, two of them showed resistant to carbapenems antibiotics, which confirmed as carbapenems resistant Enterobacteriaceae (CRE) with the prevalence of 1.83%. The carbapernems resistant bacteria increased the difficulty of antibiotic management.

**Table 4:** Comparison of drug resistance between ESBL positive and negative bacteria

Antibiotics	ESBL (+)		ESBL (–)		*P* value
	Total number	Resistant rate (*n*, %)	Total number	Resistant rate (*n*, %)	
Cefoperazone	83	48 (57.83)	44	4 (9.10)	0.000
Cefperazone-Sulbactam	84	2 (2.38)	66	0 (0.00)	0.207
Ceftriaxone	107	107 (100.00)	82	7 (8.54)	0.000
Cefmetazon	88	3 (3.40)	70	0 (0.00)	0.119
Cefepime	107	58 (54.20)	82	5 (6.10)	0.000
Cefotaxime	25	24 (96.00)	15	0 (0.00)	0.000
Ceftazidime	108	72 (66.67)	84	7 (8.33)	0.000
Levofloxacin	107	66 (61.68)	83	22 (26.51)	0.000
Gatifloxacin	24	17 (70.83)	15	5 (33.33)	0.022
Imipenem	109	2 (1.83)	83	0 (0.00)	0.215
Meropenem	103	1 (0.97)	81	0 (0.00)	0.374
Amikacin	108	3 (2.78)	81	1 (1.23)	0.466
Piperacillin	60	58 (96.67)	52	26 (50.00)	0.000
Minocycline	26	9 (34.61)	16	2 (12.50)	0.113
SMZCO	109	88 (80.73)	82	47 (57.32)	0.000
Aztreonam	107	72 (67.29)	82	4 (4.88)	0.000
Fosfomycin	68	9 (13.23)	54	1 (1.85)	0.023
Furadantin	84	9 (10.71)	66	1 (1.51)	0.025
Ticarcillin/Clavulanate Potassium	93	50 (53.76)	69	24 (34.78)	0.016
Piperacillin/tazobactam	107	7 (6.54)	84	1 (1.19)	0.067
Ampicillin	106	103 (97.17)	78	53 (67.95)	0.000

Based on their resistance to antibiotics, 27.5% of the isolated bacteria were confirmed as MDR pathogens. We investigated the association between MDR and clinical characteristics. The results suggested that ESBL status (*P* = 0.000), ICU admission (*P* = 0.005), susceptibility to the firstly selected antibiotic (*P* = 0.025), appropriate empiric therapy within 48h (*P* = 0.011), and duration of infection (*P* = 0.021) were significantly correlated with MDR. However, there were no statistic correlation between MDR and other characteristics (*P* > 0.05 for all) (Table 3).

### Risk factors for 30-day mortality based on study population

The clinical characteristics of survivors (*n* = 254) and non-survivors (*n* = 34) were compared in the study. The results demonstrated that ICU admission (*P* = 0.000), BSI sources (*P* = 0.006), hepatic encephalopathy (HE) (*P* = 0.004), pneumonia (*P* = 0.000), septic shock (*P* = 0.000), MELD at admission (*P* = 0.000), onset of BSI (*P* = 0.002), discharged (*P* = 0.000) and appropriate empiric therapy within 48 h (*P* = 0.003) were significantly different between survivors and non-survivors. In addition, other indexes did not influence the survival status (*P* > 0.05 for all) (Table 2).

**Figure 1:**
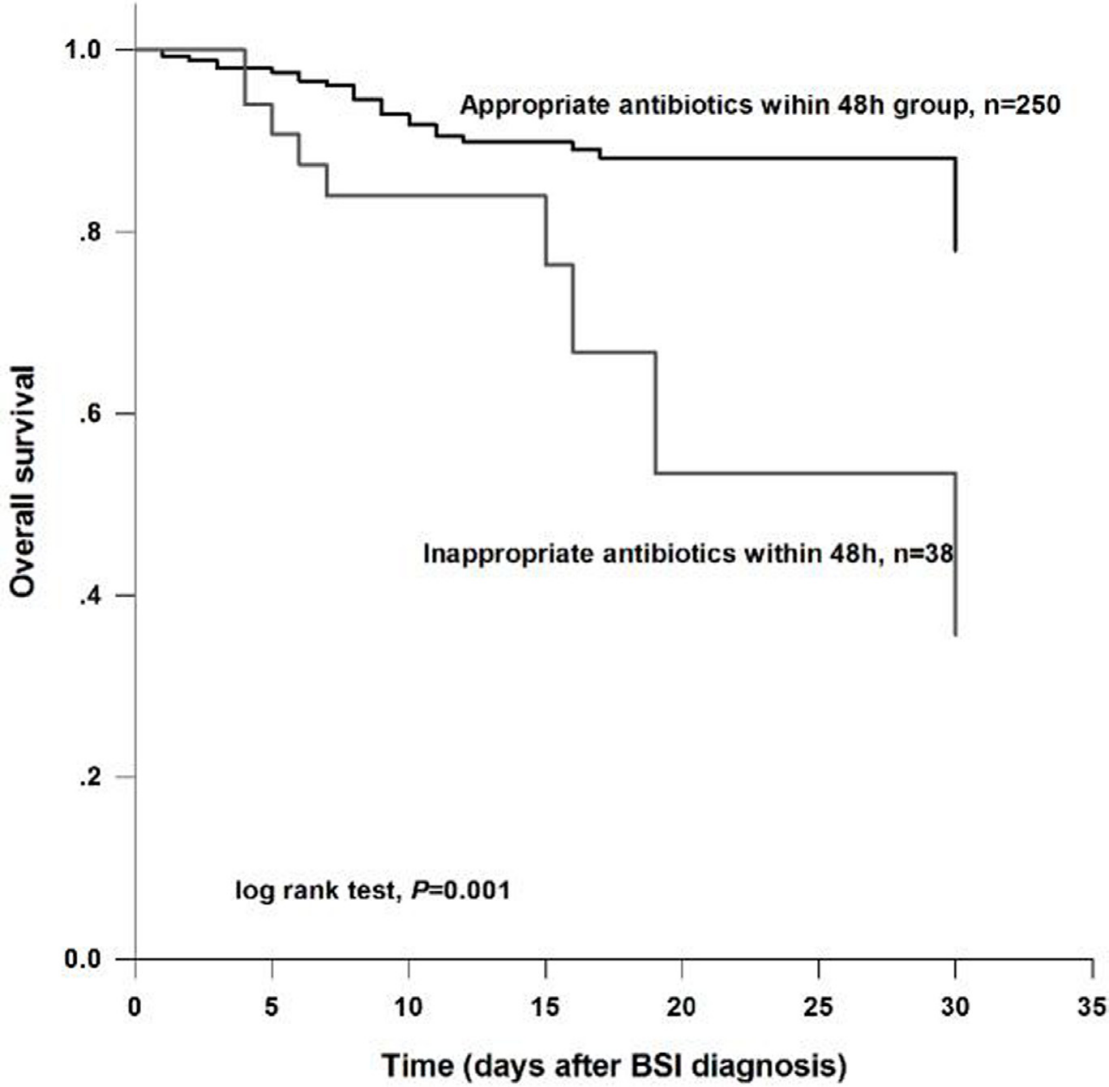
Overall survival analysis for the study subjects according to therapeutic regimens The results demonstrated that patients who received appropriate antibiotic within 48 h had a higher survival than those treated with inappropriate antibiotics within 48 h (log rank test, *P* = 0.001).

Timely and appropriate antibiotic treatments were extremely important for survival in liver cirrhosis patients developing to BSI. Survival curve demonstrated that patients received appropriate empiric therapy within 48 h after BSI diagnosis had a significantly higher survival rate than those treated with inappropriate antibiotics (log rank test, *P* = 0.001) (Figure 1).

Cox regression model was applied to evaluate the risk factors for 30-day mortality among the collected patients. Univariate analyses indicated that appropriate empiric therapy within 48 h, ICU admission, HE, pneumonia, septic shock, MELD at onset of BSI and final MELD were significantly correlated with 30-day survival rate among the study population (*P* < 0.05 for all). Multivariate analysis suggested that appropriate antibiotic treatment within 48 h (HR = 2.581, 95% CI = 1.166–5.715, *P* = 0.019), ICU admission (HR = 4.434, 95% CI = 2.130–8.823, *P* = 0.000), HE (HR = 2.379, 95% CI = 1.115–5.073, *P* = 0.025) and final MELD (HR = 1.074, 95% CI = 1.044–1.106, *P* = 0.000) were independent indicators for 30-day mortality in liver cirrhotic patients (Table 5).

**Table 5:** Risk factors for 30-day mortality in cirrhosis patients

Factors	Univariate analyses		Multivariate analyses	
	HR (95% CI)	*P*	HR (95% CI)	*P*
ESBL (positive vs negative)	0.943 (0.481–1.849)	0.864	-	-
Hospital-acquired BSI (yes vs no)	1.006 (0.507–1.994)	0.987	-	-
Sensitive to the firstly selected antibiotic (resistant vs sensitive)	1.608 (0766–3.379)	0.209	-	-
Appropriate empiric therapy within 48 h (no vs yes)	3.173 (1.514–6.647)	0.002	2.581 (1.166–5.715)	0.019
ICU admission (yes vs no)	4.578 (2.323–9.020)	0.000	4.434 (2.130–8.823)	0.000
Gender (male vs female)	0.954 (0.456–1.995)	0.900	-	-
Age	1.011 (0.982–1.041)	0.453	-	-
Combined with HCC (yes vs no)	1.239 (0.578–2.658)	0.582	-	-
HE (yes vs no)	2.320 (1.161–4.635)	0.017	2.379 (1.115–5.073)	0.025
SBP (yes vs no)	1.167 (0.594–2.294)	0.654	-	-
Pneumonia (yes vs no)	3.755 (1.750–8.054)	0.001	-	-
Child-Pugh grade (C vs A+B)	2.165 (0.979–4.785)	0.056	-	-
Septic shock (yes vs no)	3.116 (1.582–6.139)	0.001	-	-
MELD at onset of BSI	1.065 (1.019–1.114)	0.006	-	-
Final MELD	1.073 (1.044–1.102)	0.000	1.074 (1.044–1.106)	0.000

Note: ICU: Intensive care unit; HE: Hepatic encephalopathy; MELD: Model for end-stage liver diseases; Final MELD: detected at discharged; -: indicated no related data.

## DISCUSSION

Liver cirrhosis is a major healthy problems worldwide, and the critical complications resulted from cirrhosis are the leading reasons for ICU admission among the patients [22]. Liver cirrhosis can cause multiple organs failure, including liver, heart, lung, kidney, as well as immune systems [23]. Due to the functional disorder of the organs and immune system, patients with cirrhosis are susceptible to infections. BSI is a common complication for cirrhosis patients. In the present study, we evaluated the clinical characteristics and risk factors for mortality in liver cirrhosis patients with *Escherichia coli* BSI.

288 cirrhosis patients diagnosed with *Escherichia coli* BSI were collected in the present study. Among them, the occurrence rate of hospital-acquired BSI was 60.07%. We compared the clinical characteristics between hospital acquired BSI and non-hospital acquired BSI. The results suggested that patients with hospital-acquired BSI had a prolonged hospital stay. Related studies had indicated that hospital-acquired BSI could prolong hospital stay [24]. In addition, several studies indicated that hospital-acquired BSI was significantly associated with high mortality [25, 26]. However, no significant correlation between the sites of acquisition infection and mortality was found in the present study. The differences might be caused by the different medical conditions and pathogens. The effects of the sites of acquisition infection on clinical symptoms were needed to be well investigated in the next study.

ESBL production is one of the main cause for antimicrobial resistance in *Escherichia coli*, which is significantly correlated with therapeutic failures and high mortality [27, 28]. Unfortunately, the prevalence rate of ESBL positive bacteria is increasing. In the present study, 48.26% pathogens were confirmed as ESBL positive *Escherichia coli*. Drug sensitivity analysis demonstrated that these ESBL positive pathogen had a higher resistant rate to most of the common antibiotics, such as third-generation cephalosporin, quinolone. The liver cirrhosis patients infected by ESBL positive bacteria showed low sensitivity to the firstly selected antibiotics, and were more likely to undergo inappropriate empiric therapy within 48 h. Moreover, based on the drug sensitivity analysis, 27.5% pathogens were confirmed as MDR bacteria. Analysis results suggested that ESBL status, ICU admission, susceptibility to firstly selected antibiotic and appropriate empiric therapy within 48 h were significantly different between MDR infection and non-MDR infection. The result might reveal that patients infected by MDR pathogens were more likely to undergo antibiotic treatments failure and poor survival. It was worthy noting that two of the isolated bacteria were confirmed as CRE in the present study. Currently, CRE became common with high mortality. Moreover, unlike other antibiotic resistance, carapenems resistance was a complex process and mediated by several mechanisms, leading to limited therapeutic effects based on the present antibiotics [29, 30]. Related studies reported that combination antibiotic treatments could significantly reduce the mortality in patients infected with CRE [31]. The optimal antibiotic treatment regimens for CRE were needed to be investigated in further studies.

Child-Pugh score and MELD score are based on the specific characteristics of cirrhosis. These scoring standards were simple and widely applied to predict long- and short-time mortality in liver cirrhosis patients, even among those combined with infection [32]. However, accumulating evidences demonstrated that besides of the general scores, the specific parameters about infection severity should also be used to predict outcomes in liver cirrhosis patients combined with BSI [33, 34]. In the current study, Cox regression model was used to evaluate the prognostic value of clinical parameters. The results suggested that empiric therapy within 48 h, ICU admission, HE and final MELD score were independent biomarkers for prognosis in liver cirrhosis patients presenting *Escherichia coli* BSI. In the previous studies, several factors were confirmed as prognostic markers for liver cirrhosis patients combined with BSI, including MELD score, SBP as BSI source, appropriate antibiotic treatments, HE, and so on [35, 36]. All these indicators could help identify the high-risk individuals as soon as possible, thereby the therapeutic regimens were adjusted to improve clinical outcomes. It was noted that cirrhosis patients who were admitted to ICU, with high MELD or presented HE had a complex disease process and several factors might contribute to their mortality besides of infection. Therefore, novel prognostic systems combined with general and specific characteristics were needed to predict prognosis in liver cirrhosis patients combined with *Escherichia coli* BSI.

In conclusion, ESBL production is an important influencing factor for antibiotic resistance in *Escherichia coli* and patients carrying ESBL producing pathogens are more likely admitted to ICU ward, with worse MELD score. In addition, ICU admission, HE, MELD score and empiric therapy within 48h are independent predictors for outcomes in cirrhosis patients with *Escherichia coli* BSI. The present study may be useful for therapy of *Escherichia coli* BSI in patients with liver cirrhosis.

## MATERIALS AND METHODS

### Study subjects

The present study was a retrospective and observational study. The liver cirrhosis patients developing to *Escherichia coli* BSI during the period of hospitalization in Beijing 302 Hospital from May 2009 to September 2014 were collected in the study. The collected patients should meet the following criterion: (1) The study subjects were adult group; (2) All of the patients were diagnosed with decompensated liver cirrhosis; (3) All of the patients were infected with single *Escherichia coli.* And no other pathogens were isolated from the infection specimens; (4) The clinical characteristics of the patients were available. For cases who developed multiple BSIs during the study period, only the first BSI episode was included in the analysis.

### Data collection

The collected patients were pathologically diagnosed with liver cirrhosis and presented BSI during the study period. The demographics characteristics including gender and age, hospitalization information, pathogenesis and severity degree of the disease according to Child-Pugh and MELD scores [37, 38], complications during the hospital stay, baseline characteristics of BSI, and outcomes at discharged were recorded in the present study. In addition, the results of drug sensitivity test and empirical antibiotic regimen were also recorded. 30-day mortality was used to measure the primary outcomes of the collected patients. The patients developed BSI over 48 h after hospital admission were considered as hospital acquired. MELD scores were respectively recorded at the time of hospital admission, onset of infection and discharged.

### Antimicrobial susceptibility testing

Antimicrobial susceptibility of ESBL producing *Escherichia coli* isolates tested by disk diffusion method and interpreted according to Clinical Laboratories Standards Institute (CLSI) criteria [39]. The antimicrobials tested were cefoperazone, ceftriaxone, cefepime, cefotaxime, ceftazidime, levofloxacin, gatifloxacin, piperacillin, SMZCO, aztreonam, fosfomycin, furadantin, ticarcillin/clavulanate potassium potassiumand, ampicillin, cefoperazone-sulbactam, cefmetazon, meropenem, amikacin, minocycline, and piperacillin/tazobactam. *Escherichia coli* ATCC 25922 were used as negative control. The isolates that were resistant to three or more antimicrobial from different classes were defined as MDR.

### Statistical analysis

The continuous variables were presented as mean ± SD and analyzed by student’s *t* test. Chi-square test was applied to analyze the categorical variables. The clinical characteristics of the collected patients were compared according to the sites of acquisition infection, survival status, as well as the status of ESBLs of their blood cultures. Kaplan-Meier curve was performed to assess the survival of patients with different antibiotics treatment. Cox regression analysis was used to evaluate the independent risk factors for 30-day BSI mortality in cirrhotic patients. The analyses were performed with SPSS 18.0 software (SPSS Inc., Chicago, IL, USA) and *P* value less than 0.05 was considered statistically significant.

## References

[1] Liu W, Li J, Cai Y, Wu Q, Pan Y, Chen Y, Zheng X, Li W, Zhang X, E C (2016). Hepatic IGF-1R overexpression combined with the activation of GSK-3beta and FOXO3a in the development of liver cirrhosis. Life Sci.

[2] Gonzalez-Navajas JM (2016). Inflammasome activation in decompensated liver cirrhosis. World J Hepatol.

[3] Pijls KE, Smolinska A, Jonkers DM, Dallinga JW, Masclee AA, Koek GH, van Schooten FJ (2016). A profile of volatile organic compounds in exhaled air as a potential non-invasive biomarker for liver cirrhosis. Sci Rep.

[4] Karvellas CJ, Durand F, Nadim MK (2015). Acute Kidney Injury in Cirrhosis. Crit Care Clin.

[5] La Mura V, Tripodi A, Tosetti G, Cavallaro F, Chantarangkul V, Colombo M, Primignani M (2016). Resistance to thrombomodulin is associated with de novo portal vein thrombosis and low survival in patients with cirrhosis. Liver Int.

[6] Fernandez J, Gustot T (2012). Management of bacterial infections in cirrhosis. J Hepatol.

[7] Jalan R, Fernandez J, Wiest R, Schnabl B, Moreau R, Angeli P, Stadlbauer V, Gustot T, Bernardi M, Canton R, Albillos A, Lammert F, Wilmer A (2014). Bacterial infections in cirrhosis: a position statement based on the EASL Special Conference 2013. J Hepatol.

[8] Brandolini M, Corbella M, De Silvestri A, Tinelli C, Albonico G, Albertini R, Ludovisi S, Bruno R, Marone P, Minoli L, Seminari E (2015). Epidemiological characteristics of bloodstream infections in patients with different degrees of liver disease. Infection.

[9] Arvaniti V, D’Amico G, Fede G, Manousou P, Tsochatzis E, Pleguezuelo M, Burroughs AK (2010). Infections in patients with cirrhosis increase mortality four-fold and should be used in determining prognosis. Gastroenterology.

[10] Mer M (2005). Nosocomial bloodstream infection. S Afr J Infect Dis.

[11] Leber B, Spindelboeck W, Stadlbauer V (2012). Infectious complications of acute and chronic liver disease. Semin Respir Crit Care Med.

[12] Thulstrup AM, Sorensen HT, Schonheyder HC, Moller JK, Tage-Jensen U (2000). Population-based study of the risk and short-term prognosis for bacteremia in patients with liver cirrhosis. Clin Infect Dis.

[13] Bartoletti M, Giannella M, Lewis RE, Viale P (2016). Bloodstream infections in patients with liver cirrhosis. Virulence.

[14] Gunnarsdottir SA, Sadik R, Shev S, Simren M, Sjovall H, Stotzer PO, Abrahamsson H, Olsson R, Bjornsson ES (2003). Small intestinal motility disturbances and bacterial overgrowth in patients with liver cirrhosis and portal hypertension. Am J Gastroenterol.

[15] Albillos A, Lario M, Alvarez-Mon M (2014). Cirrhosis-associated immune dysfunction: distinctive features and clinical relevance. J Hepatol.

[16] Suner A, Karaoglan I, Mete AO, Namiduru M, Bosnak V, Baydar I (2015). Assessment of bloodstream infections and risk factors in an intensive care unit. Turk J Med Sci.

[17] Al-Hasan MN, Juhn YJ, Bang DW, Yang HJ, Baddour LM (2014). External validation of bloodstream infection mortality risk score in a population-based cohort. Clin Microbiol Infec.

[18] Paul M, Shani V, Muchtar E, Kariv G, Robenshtok E, Leibovici L (2010). Systematic review and meta-analysis of the efficacy of appropriate empiric antibiotic therapy for sepsis. Antimicrob Agents Ch.

[19] Retamar P, Portillo MM, Lopez-Prieto MD, Rodriguez-Lopez F, de Cueto M, Garcia MV, Gomez MJ, Del Arco A, Munoz A, Sanchez-Porto A, Torres-Tortosa M, Martin-Aspas A, Arroyo A (2012). Impact of inadequate empirical therapy on the mortality of patients with bloodstream infections: a propensity score-based analysis. Antimicrob Agents Ch.

[20] Magiorakos AP, Srinivasan A, Carey RB, Carmeli Y, Falagas ME, Giske CG, Harbarth S, Hindler JF, Kahlmeter G, Olsson-Liljequist B, Paterson DL, Rice LB, Stelling J (2012). Multidrug-resistant, extensively drug-resistant and pandrug-resistant bacteria: an international expert proposal for interim standard definitions for acquired resistance. Clin Microbiol Infec.

[21] Acevedo J (2015). Multiresistant bacterial infections in liver cirrhosis: Clinical impact and new empirical antibiotic treatment policies. World J Hepatol.

[22] Pugh RN, Murray-Lyon IM, Dawson JL, Pietroni MC, Williams R (1973). Transection of the oesophagus for bleeding oesophageal varices. The British journal of surgery.

[23] Kamath PS, Wiesner RH, Malinchoc M, Kremers W, Therneau TM, Kosberg CL, D’Amico G, Dickson ER, Kim WR (2001). A model to predict survival in patients with end-stage liver disease. Hepatology.

[24] Clinical and Laboratory Standart Institute (2012). Performance standarts for antimicrobial 335 susceptibility testing.

[25] Cho J, Choi SM, Yu SJ, Park YS, Lee CH, Lee SM, Yim JJ, Yoo CG, Kim YW, Han SK, Lee J (2016). Bleeding complications in critically ill patients with liver cirrhosis. Korean J Intern Med.

[26] Moller S, Henriksen JH, Bendtsen F (2014). Extrahepatic complications to cirrhosis and portal hypertension: haemodynamic and homeostatic aspects. World J Gastroentero.

[27] Green N, Johnson AP, Henderson KL, Muller-Pebody B, Thelwall S, Robotham JV, Sharland M, Wolkewitz M, Deeny SR (2015). Quantifying the Burden of Hospital-Acquired Bloodstream Infection in Children in England by Estimating Excess Length of Hospital Stay and Mortality Using a Multistate Analysis of Linked, Routinely Collected Data. J Pediatric Infect Dis Soc.

[28] Tsukamoto H, Higashi T, Nakamura T, Yano R, Hida Y, Muroi Y, Ikegaya S, Iwasaki H, Masada M (2014). Clinical effect of a multidisciplinary team approach to the initial treatment of patients with hospital-acquired bloodstream infections at a Japanese university hospital. Am J Infect Control.

[29] Hoenigl M, Wagner J, Raggam RB, Prueller F, Prattes J, Eigl S, Leitner E, Honigl K, Valentin T, Zollner-Schwetz I, Grisold AJ, Krause R (2014). Characteristics of hospital-acquired and community-onset blood stream infections, South-East Austria. PloS one.

[30] Alcántar-Curiel MD, Alpuche-Aranda CM, Varona-Bobadilla HJ, Gayosso-Vázquez C, Jarillo-Quijada MD, Frías-Mendivil M, Sanjuan-Padrón L, Santos-Preciado JI (2015). Risk factors for extended-spectrum beta-lactamases-producing Escherichia coli urinary tract infections in a tertiary hospital. Salud Publica Mex.

[31] Kang CI, Wi YM, Ko KS, Chung DR, Peck KR, Lee NY, Song JH (2013). Outcomes and risk factors for mortality in community-onset bacteremia caused by extended-spectrum beta-lactamase-producing Escherichia coli, with a special emphasis on antimicrobial therapy. Scand J Infect Dis.

[32] Jacob JT, Klein EY, Ramanan L, Beldavs ZG, Lynfield R, Kallen AJ, Ricks P, Edwards JR, Srinivasan A, Fridkin SK, Rasheed JK, Lonsway D, Bulens SN (2013). Vital signs: carbapenem-resistant Enterobacteriaceae. MMWR Morb Mortal Wkly Rep.

[33] O’Horo JC, Farrell A, Sohail MR, Safdar N (2016). Carbapenem-resistant Enterobacteriaceae and endoscopy: An evolving threat. Am J Infect Control.

[34] Garbati MA, Sakkijha H, Abushaheen A (2016). Infections due to Carbapenem Resistant Enterobacteriaceae among Saudi Arabian Hospitalized Patients: A Matched Case-Control Study. Biomed Res Int.

[35] Durand F, Valla D (2008). Assessment of prognosis of cirrhosis. Semin Liver Dis.

[36] Viasus D, Garcia-Vidal C, Castellote J, Adamuz J, Verdaguer R, Dorca J, Manresa F, Gudiol F, Carratala J (2011). Community-acquired pneumonia in patients with liver cirrhosis: clinical features, outcomes, and usefulness of severity scores. Medicine.

[37] Thabut D, Massard J, Gangloff A, Carbonell N, Francoz C, Nguyen-Khac E, Duhamel C, Lebrec D, Poynard T, Moreau R (2007). Model for end-stage liver disease score and systemic inflammatory response are major prognostic factors in patients with cirrhosis and acute functional renal failure. Hepatology.

[38] Bartoletti M, Giannella M, Caraceni P, Domenicali M, Ambretti S, Tedeschi S, Verucchi G, Badia L, Lewis RE, Bernardi M, Viale P (2014). Epidemiology and outcomes of bloodstream infection in patients with cirrhosis. J Hepatol.

[39] Jepsen P, Ott P, Andersen PK, Sorensen HT, Vilstrup H (2010). Clinical course of alcoholic liver cirrhosis: a Danish population-based cohort study. Hepatology.

